# Stochastic Recursive Gradient Support Pursuit and Its Sparse Representation Applications

**DOI:** 10.3390/s20174902

**Published:** 2020-08-30

**Authors:** Fanhua Shang, Bingkun Wei, Yuanyuan Liu, Hongying Liu, Shuang Wang, Licheng Jiao

**Affiliations:** Key Laboratory of Intelligent Perception and Image Understanding of Ministry of Education, School of Artificial Intelligence, Xidian University, Xi’an 710071, China; bkwei028@gmail.com (B.W.); yyliu@xidian.edu.cn (Y.L.); hyliu@xidian.edu.cn (H.L.); shwang@mail.xidian.edu.cn (S.W.); lchjiao@mail.xidian.edu.cn (L.J.)

**Keywords:** sparse learning, hard thresholding, stochastic optimization, variance reduction

## Abstract

In recent years, a series of matching pursuit and hard thresholding algorithms have been proposed to solve the sparse representation problem with $\ell_{0}$-norm constraint. In addition, some stochastic hard thresholding methods were also proposed, such as stochastic gradient hard thresholding (SG-HT) and stochastic variance reduced gradient hard thresholding (SVRGHT). However, each iteration of all the algorithms requires one hard thresholding operation, which leads to a high per-iteration complexity and slow convergence, especially for high-dimensional problems. To address this issue, we propose a new stochastic recursive gradient support pursuit (SRGSP) algorithm, in which only one hard thresholding operation is required in each outer-iteration. Thus, SRGSP has a significantly lower computational complexity than existing methods such as SG-HT and SVRGHT. Moreover, we also provide the convergence analysis of SRGSP, which shows that SRGSP attains a linear convergence rate. Our experimental results on large-scale synthetic and real-world datasets verify that SRGSP outperforms state-of-the-art related methods for tackling various sparse representation problems. Moreover, we conduct many experiments on two real-world sparse representation applications such as image denoising and face recognition, and all the results also validate that our SRGSP algorithm obtains much better performance than other sparse representation learning optimization methods in terms of PSNR and recognition rates.

## 1. Introduction

In recent years, sparse representation has been proved to be a useful approach to represent or compress high dimensional signals. Sparse representation algorithms have attracted many researchers in the fields of signal processing, image processing, medical imaging, machine learning, computer vision, pattern recognition, and so on [1]. In most applications, the unknown signal of interest is regarded as a sparse combination of a few columns from a given dictionary, and this problem is usually formulated as a sparsity constrained problem. Such sparse representation problems are common in the fields of image denoising, image inpainting, and face recognition or others such as [2,3,4].

Image denoising is a classical problem to improve image quality in computer vision. The aim of this problem is to recover the clean image x from the noisy image y=x+e, where e is additive white Gaussian noise in general [5]. It can be realized generally by the following three types of methods: transform domain [6], spatial filtering [7], and dictionary learning-based methods [8,9]. Note that the dictionary learning-based methods optimize the following model with the step of $\ell_{0}$-norm sparse coding:

(1) $$\min_{x\in R^{d}}{∥x∥}_{0},\;\;\;s.t.,{∥y-Dx∥}_{2}^{2}\leq \varepsilon ,$$

where x represents the sparse coding of $y\;\in \;R^{n}$ with a given error tolerance ε>0, $D\;\in \;R^{n\times d}$ is a given dictionary, ${∥x∥}_{0}$ denotes the number of nonzero entities of the vector x, and ${∥x∥}_{2}$ is the $\ell_{2}$-norm, i.e., ${∥x∥}_{2}\;=\;\sqrt{\sum_{i}x_{i}^{2}}$. This is an $\ell_{0}$-norm constrained minimization problem, which can be solved via convex relaxation or greedy algorithms. The performance of the algorithms for finding a sparse representation solution has a big impact on denoised images. Similar effects can be found in face recognition tasks.

As a challenging application in computer vision and pattern recognition, face recognition has become a more complicated problem on account of illuminations, occlusions, expressions, and facial disguises. Many face recognition methods have been proposed, e.g., Eigenfaces [10] and Fisherfaces [11]. Wright et al. [12] proposed a sparse representation-based classification (SRC) method, which regards the training face images themselves as an overcomplete dictionary. It is possible for a testing face image to be represented by the atoms of the overcomplete dictionary, and thus we need to solve a sparse coding problem similar to Equation (1). Hence, there is the same question to image denoising. In this paper, our main aim is to find a more efficient algorithm for sparse representation on image denoising and face recognition tasks.

### 1.1. Stochastic Hard Thresholding Methods

So far, there have been many algorithms for pursuing sparse solutions using $\ell_{p}$-norm (0≤p≤1) minimization [1]. Although convex relaxation algorithms are easy to find an optimal solution due to the convexity of $\ell_{1}$-norm, $\ell_{1}$-norm minimization has some limits as pointed out in [13] and sometime obtains worse empirical performance [14]. Thus, it is necessarily to solve the $\ell_{0}$-norm problem directly. In order to obtain the sparse solutions, many greedy algorithms [15,16,17,18,19] have been developed. Moreover, there are some hard thresholding-based methods, such as iterative hard thresholding [20], fast gradient hard thresholding pursuit [21], and gradient support pursuit (GraSP) [22]. All the methods have many successful applications for various real-world problems such as sparse vector and low-rank matrix recovery. However, the hard thresholding methods are deterministic optimization algorithms, they need to compute a full gradient using all training samples and have a high per-iteration complexity O(nd), which makes them unsuitable for real-world large-scale data.

To address this issue, Nguyen et al. [23] proposed a stochastic gradient hard thresholding (SG-HT) algorithm, and introduced the idea of stochastic optimization into hard thresholding methods. It randomly selects one sample to optimize per-iteration and holds a much lower complexity. However, SG-HT cannot decrease the variance between the stochastic gradient and its full gradient. Li et al. [24] proposed a stochastic variance reduced gradient hard thresholding (SVRGHT) method, which uses the stochastic variance reduction gradient (SVRG) technique [25] as well as in [26]. With the help of variance reduction techniques, SVRGHT can obtain a faster convergence rate. More recently, there have been several stochastic hard thresholding algorithms using first-order or second-order information [27,28,29,30,31,32,33]. However, many stochastic algorithms such as SVRGHT have a hard thresholding operation in each iteration, whose computational complexity is relatively high O(dlog(d)) in general [34], especially for high-dimensional data. In addition, there are two main drawbacks for the thresholding methods. The first shortcoming is the optimization theoretical basis. That is, when the current iterate solution is not a minimizer of the function, moving from the solution in the direction of negative gradient of the function leads to the decrease in the value of this function. However, this assumption is not generally true when the hard thresholding operator $H_{s}\left(\cdot \right)$ is applied to the current vector $x^{t}$, which means that the gradient information has lost. It breaks the information of the current solution and may waste much computation to perform gradient descent. Secondly, the computational burden of hard thresholding operation is still linear with *d*, which can not be negligible. There exists an interesting question whether there is an algorithm to overcome these drawbacks. We answer this question affirmatively in theory and in practice.

### 1.2. Our Contributions

In this paper, we propose the first variance reduced stochastic recursive gradient method for sparse representation problems. In other words, we use the stochastic recursive gradient proposed in [35], which is suitable for solving non-convex problems, to optimize the non-convex sparse representation problem in this paper. In order to keep the gradient information of current iterate as suggested in [36], we perform lots of gradient descent steps, followed by a hard thresholding operation. We also construct the most relevant support on which minimization will be efficient. Therefore, this paper proposes a novel sparsity-constrained algorithm, called stochastic recursive gradient support pursuit (SRGSP). At each iteration in SRGSP, we first find the most relevant support set, minimize slackly over the support set by our stochastic recursive gradient solver, which satisfies a certain descent condition, and then perform hard thresholding on the updated model parameter. The main contributions and novelty of this paper are listed as follows:

(1) It is is non-trivial that we analyze the statistical estimation performance of SRGSP under mild assumptions, and the theoretical results show that SRGSP obtains a fast linear convergence rate.
(2) Benefiting from less hard thresholding operations than existing algorithms such as SVRGHT, the average per-iteration cost of our algorithm is much lower (O(d) for SRGSP vs. O(dlog(d)) for SVRGHT), which leads to faster convergence.
(3) Moreover, less usage of hard thresholding operators to the current variable results in retain of gradient optimization information, which improves empirical performances. Stochastic recursive gradient support pursuit leads to a new trend to reduce the complexity of head thresholding operation while maintaining or even improving the performance.
(4) We also evaluate the empirical performance of our SRGSP method on sparse linear and logistic regression tasks as well as real-world applications such as image denoising and face recognition. Our experimental results show the efficiency and effectiveness of SRGSP.

The remainder of this paper is organized as follows. In Section 2, we introduce the related applications (i.e., image denoising and face recognition), and we propose our SRGSP algorithm in Section 3. The convergence analysis is provided in Section 4. In Section 5, many experimental results on both synthetic and real-world datasets verify the effectiveness of SRGSP, and the results of image denoising and face recognition further demonstrate the superiority of SRGSP against some state-of-the-art hard thresholding algorithms. Section 6 presents conclusions and future work.

## 2. Related Work

In this section, we start with a brief description of some related applications, in which sparse representation can play an important role.

### 2.1. Notation

In this paper, ${∥x∥}_{0}$ denotes the number of nonzero entities of the vector x, supp(x) denotes the index set of nonzero entities of x, and supp(x,s) is the index set of the top *s* entries of x in terms of magnitude. In addition, we denote $I^{c}$ the complement set of *I* and ${x|}_{I}$ the restriction of vector x to the rows indicated by indices in *I*. Furthermore, we denote $H_{F}\left(\cdot \right)$ the Hessian matrix of the function F(·), and denote E(·) the expectation.

### 2.2. Sparse Representation-Based Image Denoising

In sparse representation, the clean images or signals can be approximated via a sparse combination of coefficients from a basis set, called dictionary. In this circumstance, denoising a patch vector $y^{j}$, which is extracted from the noisy image matrix, with a dictionary $D\in R^{n\times d}$ is regarded as solving the following sparsity constrained optimization problem:

(2) $$\min_{x}\;F\left(x\right)\;\overset{\mathrm{def}}{=}\frac{1}{n}\sum_{i=1}^{n}f_{i}\left(x\right)\;=\;\frac{1}{n}\sum_{i=1}^{n}∥y_{i}^{j}-D_{i}{x∥}_{2}^{2},\;s.t.,\;{∥x∥}_{0}\leq s,$$

where $D_{i}x$ is an estimate of $y_{i}^{j}$, *s* is a sparsity constant, and $y^{j}$ is the *j*-th patch of the noisy image *y*. There are many dictionary learning algorithms such as [8,37,38], which alternately update the dictionary with learned sparse iterate x. Although these algorithms have demonstrated that learned dictionaries on noisy images or on a set of good quality images can achieve better performance than off-the-shelf ones such as [9], we here use the fixed overcomplete dictionary for verifying the property of our algorithm in sparse coding. The overcomplete dictionary D, which means that the number of columns may be greater than the number of rows, can be obtained by the discrete cosine transform (DCT) [39] or its redundant version, as implemented in [38]. Since general images may be very large, current practices sparsely represent image patches rather than the full image.

In summary, we obtain an overcomplete dictionary matrix D by DCT and then use $\ell_{0}$-norm constrained optimization algorithms to find an approximate solution of Equation (2) to restore the image.

### 2.3. Sparse Representation-Based Face Recognition

Face recognition is an active research field in computer vision, and this task is to use *k* classes labeled training samples to classify the testing sample into the correct class. In this paper, we take our algorithm into the SRC framework [12] for face recognition. As SRC uses $\ell_{1}$-norm minimization to solve the sparse coding model, in this paper we use $\ell_{0}$-norm minimization instead, which can also obtain a sparse solution and this algorithm is provided in the Appendix A. In the SRC algorithm, an l×h gray facial training image is reshaped into a column vector $a_{r,u}\;\in \;R^{n}$, i.e., n=lh. Then we construct the matrix $A_{r}\;=\;\left[a_{r,1},a_{r,2},\cdots ,a_{r,d_{r}}\right]\;\in \;R^{n\times d_{r}}$ by using $d_{r}$ training samples belonging to the *r*-th class. For each testing sample $y^{r}={\left[y_{1}^{r},\cdots ,y_{n}^{r}\right]}^{⊤}\in R^{n\times 1}$ in the same class can be linear represented by the columns in $A_{r}$:

(3) $$y^{r}\;=\;a_{r,1}x_{r,1}+a_{r,2}x_{r,2}+\cdots +a_{r,d_{r}}x_{r,d_{r}}.$$

Here, $x_{r,1},x_{r,2},\cdots ,x_{r,d_{r}}$ are all scalars, which are the representation coefficients for $y^{r}$. Since the testing sample is unknown, then we consider all training samples of *k* classes and define a matrix *A*: $A\;=\;\left[A_{1},A_{2},\cdots ,A_{k}\right]\;\in \;R^{n\times d}$. Therefore, the representation of a testing sample can be rewritten with respect to all the training samples as:

(4) $$y^{r}=Ax_{0},$$

where $x_{0}$ is a coefficient vector, whose nonzero elements only associated with the *r*-th class. In this paper, sparse representation with $\ell_{0}$-norm minimization can be used to solve the following sparsity constrained optimization problem in the SRC framework:

(5) $$\min_{x}\;F\left(x\right)\;=\;\frac{1}{n}\sum_{i=1}^{n}f_{i}\left(x\right)=\frac{1}{n}\sum_{i=1}^{n}∥y_{i}^{r}-A_{row}^{i}{x∥}_{2}^{2},\;s.t.,\;{∥x∥}_{0}\leq s,$$

where *s* is the sparse constant, which implies the number of nonzero elements of x and $A_{row}^{i}\in R^{1\times d}$ is the *i*-th row vector of *A*. Defining $\delta_{p}:R^{d}\;\to \;R^{d}$ is a selection function corresponding to the *p*-th class. Given a sparse vector $x\;\in \;R^{d}$ from Equation (5), $\delta_{p}\left(x\right)\;\in \;R^{d}$ is a new vector, whose nonzero elements are only associated with the *p*-th class. Then minimizing the following residual function can encode the identity of the sample $y^{r}$ as follows:

(6) $$\mathrm{identity}\left(y^{r}\right)=\arg\min_{p}\;\left\{R_{r}\left(y\right):={∥y^{r}-A\delta_{p}\;\left(x\right)∥}_{2}\right\}.$$

## 3. Our Stochastic Recursive Gradient Support Pursuit Method

In this section, we propose a novel stochastic recursive gradient support pursuit (SRGSP) method for sparsity constrained problems. Different from existing gradient support pursuit methods (e.g., GraSP [22]), SRGSP only requires to satisfy a certain constrictive condition in each iteration, and thus has a faster convergence speed in practice.

In recent years, many non-convex gradient support pursuit methods such as [20,22] have been proposed, and it has also been shown that they can have better performance than convex $\ell_{1}$-norm methods in certain circumstances. Most of the existing gradient support pursuit algorithms use deterministic optimization methods to minimize various sparse learning problems (e.g., Problem (2)). However, the per-iteration complexity of all these algorithms is O(nd), which leads to slow convergence, especially for large-scale and high-dimensional problems. Inspired by GraSP [22], which is a well-known gradient support pursuit method, we propose an efficient stochastic recursive gradient support pursuit (SRGSP) algorithm to approximate the solution to Problem (2), as outlined in Algorithm 1.
**Algorithm 1**: Stochastic Recursive Gradient Support Pursuit (SRGSP)**Input:** Sparsity level *s*, learning rate η, the numbers of outer-iterations and inter-iterations, *T* and *J*.**Initialize:** ${\hat{x}}^{0}$.  1:**for**t=1,2,⋯,T**do**2: Compute current gradient: $g^{0}=\nabla F({\hat{x}}^{t-1})$;   3: Identify directions: $Z=\mathrm{supp}(g^{0},2s)$;   4: Merge supports: $T=Z\cup \mathrm{supp}({\hat{x}}^{t-1})$;   5: Initialization: $z^{0}={\hat{x}}^{t-1}$, $z^{1}\;=z^{0}\;-\eta g^{0}$;   6: **for** j=1,2,⋯,J **do**
7:  Randomly pick $i_{j}\;\in \;\left\{1,2,\ldots ,n\right\}$;   8:  $g^{j}\;=\nabla \;f_{i_{j}}\;\left(z^{j}\right)-\;\nabla \;f_{i_{j}}\;\left(z^{j-1}\right)+g^{j-1}$;   9:  $z^{j+1}\;=z^{j}\;-\eta g^{j}$;   10: **end for**  11: Perform hard thresholding over  T: ${\hat{x}}^{t}\;=H_{s}\left(z^{J+1}\;{|}_{T}\right)$;   12:**end for****Output:** ${\hat{x}}^{T}$.

At each iteration of Algorithm 1, we first compute the gradient of F(·) at the current estimate, i.e., $g^{0}\;=\;\nabla F\left({\hat{x}}^{t-1}\right)$. Then we choose 2s coordinates of $g^{0}$ that have the largest magnitude as the direction in which pursuing the minimization will be most effective, and denote their indices by Z, where *s* is the sparsity constant. Merging the support of the current estimate with the 2s coordinates mentioned above, we can obtain the combined support, which is a set of at most 3s indices, i.e., $T\;=\;Z\;\cup \;\mathrm{supp}({\hat{x}}^{t-1})$ (Some parameters used in this paper have already been defined in the second section). Over the current support set T, we compute an estimate b by using stochastic recursive gradient descent as the approximate solution to the problem (7).

The key difference between GraSP and SRGSP is that GraSP needs to yield the exact solution $\hat{b}$ to the following minimization problem:

(7) $$\min_{x}\;F\left(x\right),\;\;s.t.,\;\;x{|}_{T^{c}}=0,$$

where $T^{c}$ is the complement set of T in line 4 of Algorithm 1, while our SRGSP method only requires a sub-solver (e.g., the iteration steps from Step 5 to Step 10 in Algorithm 1), which is to find an approximate solution b to Problem (7) satisfying

(8) $$∥b-\hat{b}{∥}_{2}\leq c_{1}{∥{\hat{x}}^{t-1}-\hat{b}∥}_{2},$$

where $\hat{b}$ is the exact solution to Equation (7), ${\hat{x}}^{t-1}$ is the temporary result of last outer-iteration, $0\;<\;c_{1}\;<\;1$ is an error bound constant, meaning that our algorithm can have a guaranteed decrease at each iteration, as shown in our convergence analysis in the next section. In other words, we can select other efficient solvers (e.g., SVRG [25], VR-SGD [32] and their accelerated variants [40,41]) for the proposed framework, as long as the solvers satisfy the certain constrictive condition in Equation (8). Since stochastic recursive gradient descent in [42,43] has been proved to have a faster convergence rate than other stochastic gradient operators such as SVRG [25] for solving non-convex optimization problems, we choose the former as our solver rather than SVRG as in [24]. When a fully deterministic optimization method is used as a sub-solver in Algorithm 1 for solving Problem (7), GraSP can be viewed as a special case of SRGSP.

In our experiments, we usually set J=2n as the number of iterations similar to the original SARAH algorithm [35]. Within each inner-loop of Algorithm 1, our main update rules are as follows:

$$g^{j}=\nabla \;f_{i_{j}}\;\left(z^{j}\right)-\nabla \;f_{i_{j}}\;\left(z^{j-1}\right)+g^{j-1},$$

$$z^{j+1}=z^{j}\;-\eta g^{j}.$$

Note that $g^{j}$ is the stochastic recursive gradient, which is first proposed in [35]. That is, our  SRGSP algorithm updates $g^{j}$ using the accumulated stochastic information, which has the advantage of accelerating convergence naturally. The parameter ${\hat{x}}^{t}$ is then updated using the hard thresholding operator, which keeps the largest *s* terms of the intermediate estimate b. This step makes ${\hat{x}}^{t}$ as the best *s*-term approximation of the estimate b. The hard thresholding operator is defined as follows:

$${\left[H_{s}\left(x\right)\right]}_{i}=\left\{\begin{array}{ccc} & x_{i}, & if\;i\in supp(x,s),\\ & 0, & otherwise, \end{array}\right.$$

where $x_{i}$ is the *i*-th coordinate value of the vector x.

**Assumption** **1.** *The solution to the sub-problem (7) is unique.*

From the above analysis, we can find that our SRGSP algorithm uses a hard thresholding operation after lots of stochastic recursive gradient updates, while existing stochastic algorithms such as SVRGHT [24] perform hard thresholding in each inner-iteration, which is very time consuming for high-dimensional problems.

## 4. Convergence Analysis

In this section, we provide the convergence analysis of our SRGSP algorithm.

### 4.1. Convergence Property of Our Sub-solver

In this part, we consider the convergence property of our sub-solver in Algorithm 1, that is, our sub-solver can satisfy the descent condition in Equation (8). As most of the algorithms available in the community provide the bound for $∥F\left(b\right)\;-\;F\left(\hat{b}\right){∥}_{2}$, our convergence analysis requires other structures in F(·) to obtain a bound for $∥b\;-\;\hat{b}{∥}_{2}$. Therefore, we would like to introduce the following insightful summary of the structures of F(·) [44].

**Lemma** **1.** *Let F(·) be a function with a Lipschitz-continuous gradient, the following implications hold:*

$$\begin{array}{c} (SC)\to (ESC)\to (WSC)\to (PL)\to (QG), \end{array}$$

*where SC means Strong Convexity, ESC means Essential Strong Convexity, WSC means Weak Strong Convexity, PL means Polyak–Lojasiewicz, and QG means Quadratic Growth. For their definitions, we would refer the reader to [44]. If we further assume that F(·) is convex, then we have (PL)≡(QG).*

These results show that QG is the weakest assumption. Next, we prove that our sub-solver satisfies the descent condition in Equation (8).

**Theorem** **1.** *Suppose F(·) satisfies the QG-condition with the parameter ρ and is Lipschitz continuous with the parameter L. Assume that the number of inter-iterations, J, is sufficiently large, our sub-solver has the following expected convergence property:*

(9) $$\begin{array}{c} E\left[F\left(b\right)-F\left(\hat{b}\right)\right]\leq c_{2}\left[F\left({\hat{x}}^{t-1}\right)-F\left(\hat{b}\right)\right], \end{array}$$

*where $0\;<\;c_{2}\;<\;1$ is a constant, and then we have*

$$\begin{array}{c} ∥b-\hat{b}{∥}_{2}\leq \frac{2c_{2}L}{\rho }{∥{\hat{x}}^{t-1}-\hat{b}∥}_{2}. \end{array}$$

The detailed proofs of Theorem 1 and the theorem below are provided in the Supplementary Material. Similar to the linear convergence analysis of SARAH for solving convex problems in [35], our sub-solver can exhibit expected descent in the objective function value, as shown in Equation (9). If our sub-solver with a sufficiently large number of inter-iterations, then $c_{2}$ can be a very small constant. According to Theorem 1, one can easily verify that our sub-solver can satisfy the constrictive condition in Equation (8) when $\frac{2c_{2}L}{\rho }\;\leq \;c_{1}$. That is, our sub-solver with a sufficiently large number of inter-iterations can satisfy the constrictive condition in Algorithm 1.

### 4.2. Convergence Property of SRGSP

Before giving our main convergence result, we first present some important definitions.

**Definition** **1** (Stable Restricted Hessian). *Suppose that F(·) is a twice continuously differentiable function, and its Hessian matrix is denoted by $H_{F}\left(\cdot \right)$. For a given positive integer k, let*

(10) $$\begin{array}{cc} A_{k}\left(u\right) & =\underset{\left|\mathrm{supp}\left(u\right)⋃\mathrm{supp}\left(v\right)\right|\leq {k,∥v∥}_{2}=1}{\sup}v^{T}H_{F}\left(u\right)v, \end{array}$$

(11) $$\begin{array}{cc} B_{k}\left(u\right) & =\underset{\left|\mathrm{supp}\left(u\right)⋃\mathrm{supp}\left(v\right)\right|\leq {k,∥v∥}_{2}=1}{\inf}v^{T}H_{F}\left(u\right)v, \end{array}$$

*for all k-sparse vectors u. Then F(·) is said to have a Stable Hessian Property (SRH) with constant $\mu_{k}$, or in short $\mu_{k}$-SRH, if $1\leq \frac{A_{k}\left(u\right)}{B_{k}\left(u\right)}\leq \mu_{k}$.*

This definition shows that the SRH condition is similar to various forms of Restricted Strong Convexity (RSC) used in the performance analysis of existing sparsity constrained algorithms [22]. Note that this property is suitable for smooth loss functions, and there are a broad family of loss functions that have Lipschitz-continuous gradients.

**Theorem** **2.** *Let F(·) be a twice continuously differentiable function that has $\mu_{4s}$-SRH with $\mu_{4s}\;<\;\sqrt{2}$, and satisfies Assumption 1. For some ϵ>0, we have $\epsilon \;<\;B_{4s}\left(u\right)$ for all 4s-sparse u, and $\left\{{\hat{x}}^{t}\right\}$ is a sequence generated by Algorithm 1. Then we have*

$$\begin{array}{cc} ∥{\hat{x}}^{t}-x^{*}{∥}_{2}\leq & \;\delta^{t}∥{\hat{x}}^{0}-x^{*}{∥}_{2}+\left[1\;-\;\delta^{t}\right]\frac{\left(1\;+\;c_{1}\right)\left(2\mu_{4s}\;+\;4\right)}{\epsilon (1-\delta )}∥\nabla \;F\left(x^{*}\right){{|}_{I}∥}_{2}, \end{array}$$

where $\delta :=\left(1\;+\;c_{1}\right)\left(\mu_{4s}^{2}\;-\;1\right)+2c_{1}<1$, and I is the position of the 3s largest entries of $\nabla \;F(x^{*})$ in magnitude.

As discussed above, our sub-solver with a sufficiently large number of inter-iterations can satisfy the constrictive condition in Equation (8) with a very small constant $c_{1}$, which makes δ<1 hold. Then we also have $0\;<\;c_{1}\;<\;\frac{2-\mu_{4s}^{2}}{1+\mu_{4s}^{2}}$. This implies that our sub-solver has to achieve a certain accuracy for the theorem to work. Theorem 2 suggests that our proposed algorithm achieves a linear convergence rate. This error bound consists of two terms, where the first term corresponds to the optimization error and the second term corresponds to the statistical error. After sufficient iterations, the second term will approach to zero. Therefore, our algorithm can always converge to the unknown true parameter $x^{*}$ with increasing of iterations.

## 5. Experimental Results

In this section, we evaluate the performance of our SRGSP method on synthetic and real-world large-scale datasets. Moreover, we apply SRGSP to tackle various sparse representation problems including image denoising and face recognition tasks. In this work, we only use the two real-world applications to illustrate the excellent performance of our SRGSP algorithm against other sparse learning optimization methods including GraSP [22], SG-HT [23], SVRGHT [24], and loopless semi-stochastic gradient descent with less hard thresholding (LSSG-HT) [34].

### 5.1. Baseline Methods

We compared the proposed algorithm (i.e., SRGSP) with four state-of-the-art algorithms: gradient support pursuit (GraSP) [22], stochastic gradient descent with hard thresholding (SG-HT) [23], stochastic variance reduced gradient with hard thresholding (SVRGHT) [24] and loopless semi-stochastic gradient descent with less hard thresholding (LSSG-HT) [34].

### 5.2. Synthetic Data

We generated a synthetic matrix *A* with size n×d, each row of which is drawn independently from a *d*-dimensional Gaussian distribution with mean 0 and covariance matrix $\Sigma \;\in \;R^{d\times d}$. The response vector was generated from the linear model $y\;=\;Ax^{*}\;+e$, where $x^{*}\;\in \;R^{d}$ is the $s^{*}$-sparse coefficient vector, and the noise e was generated from a multivariate normal distribution $N(0,\sigma^{2}I)$ with $\sigma^{2}=0.01$. The nonzero entries in $x^{*}$ were sampled independently from a uniform distribution over the interval [−1,1]. For the experiments, we constructed two synthetic data: (1)$n\;=\;2500,\;d\;=\;5000,\;s^{*}\;=\;250,\;\Sigma \;=\;I$; (2) $n\;=\;5000,\;d\;=\;10,000,\;s^{*}\;=500$ and the diagonal entries of the covariance matrix Σ were set to 1, and the other entries were set to 0.1. The sparsity parameter *s* was set to $s=1.2s^{*}$ for all the algorithms.

**Figure 1:**
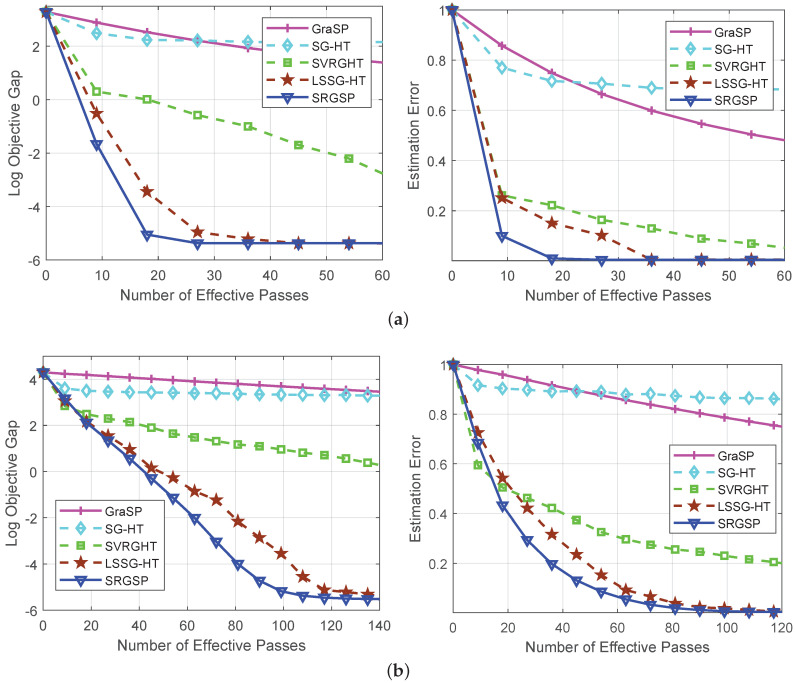
Comparison of gradient support pursuit (GraSP) [22], gradient support pursuit (SG-HT) [23], stochastic variance reduced gradient with hard thresholding (SVRGHT) [24], loopless semi-stochastic gradient descent with less hard thresholding (LSSG-HT) [34] and SRGSP for solving sparse linear regression problems on synthetic data. (**a**) $n\;=\;2500,d\;=\;5000,s^{*}\;=\;250$; (**b**) n=5000, d=10,000, $s^{*}\;=\;500$.

Figure 1 shows the performance (including the logarithm of the objective function values and the estimation error $\frac{∥{\hat{x}}^{t}-x^{*}{∥}_{2}}{∥x^{*}{∥}_{2}}$) of all the algorithms on the synthetic data. All the results show that our algorithm converges significantly faster than the state-of-the-art methods in terms of function values and estimation error in all the settings. Although our SRGSP algorithm, SVRGHT and LSSG-HT have been theoretically proved to have a linear convergence rate, SRGSP consistently outperforms SVRGHT and LSSG-HT in terms of number of effective passes.

### 5.3. Real-World Data

In this subsection, we focus on the two real-world large-scale datasets: rcv1-train and real-sim, which can be downloaded from the LIBSVM website (https://www.csie.ntu.edu.tw/~cjlin/libsvm/). In our experiments, we use the rcv1-train and real-sim datasets to evaluate the performance for linear regression, and the two datasets include 20,242 samples with 47,236 features and 72,309 samples with 20,958 features, respectively. Moreover, we choose s=200 for the two datasets and compare all the algorithms in terms of logarithm objective value gap versus the number of effective passes and running time (in seconds).

**Figure 2:**
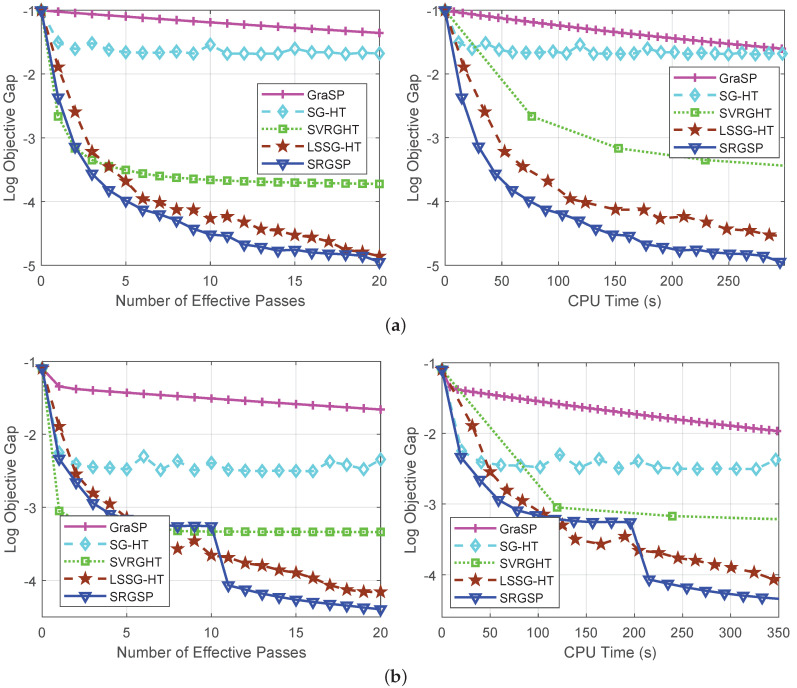
Comparison of GraSP [22], SG-HT [23], SVRGHT [24], LSSG-HT [34] and our SRGSP method for solving sparse linear regression problems. In each plot, the vertical axis shows the logarithm of the objective value minus the minimum, and the horizontal axis is the number of effective passes over data or running time (in seconds). (**a**) rcv1; (**b**) real-sim.

Figure 2 illustrates the performance of our algorithm in terms of the logarithm of function gap (i.e., $\log(∥F\left({\hat{x}}^{t}\right)\;-\;F\left(x^{*}\right){∥}_{2})$). More specifically, our SRGSP algorithm has a faster convergence rate than the four state-of-the-art sparsity constrained algorithms. In addition, SRGSP has the ability to jump out of a local minimum and can find a better solution, as shown in Figure 2b. Compared with SVRGHT, we can see that the results of our SRGSP in the first few iterations are similar to SVRGHT. However, due to lots of gradient updates followed by a hard thresholding, SRGSP can obtain a better solution, as discussed in [36]. This further verify the advantage of our SRGSP against other methods. On the other hand, our SRGSP algorithm can reach better solutions in much less CPU time than the other methods, including SVRGHT and LSSG-HT. Since SRGSP has one hard thresholding operation per iteration, while SVRGHT needs *n* operations (*n* is the number of samples) in each epoch, and thus SVRGHT has a higher per-iteration complexity than SRGSP, especially for large-scale and high-dimensional data. Therefore, our SRGSP algorithm is very suitable for the large-scale non-convex sparsity constrained problem.

### 5.4. Image Denoising

In this subsection, we apply our SRGSP algorithm to image denoising tasks for evaluating its performance. First of all, the most important of image denoising is to find a suitable dictionary. In fact, the DCT seems like a reliable choice following the Guleryuz’s work [45]. We use the overcomplete DCT dictionary in our experiments. Similar to [8], the error bound ε is set empirically as 1.15σ. The experiments are conducted on 6 standard benchmark images with synthetic white Gaussian noise. The sparsity level parameter in this experiment is set as s=10 and the Gaussian random noise with zero mean and standard deviation =σ is added to the standard images. The dictionary in the experiments is the fixed overcomplete DCT dictionary of size 64×256, and is designed to deal with image patches of 8×8 pixels. The denoising processes are mainly concentrated on sparse coding of these patches using different sparsity constrained algorithms (e.g., GraSP, SG-HT, SVRGHT, LSSG-HT and SRGSP) and the classical greedy orthogonal matching pursuit (OMP) algorithm [46]. The parameters of x are computed till the loss of Equation (1) lower than the error bound or the number of iterations larger than 32 (half of the row size of dictionary). When computing the sparse representative solution on overlapping patches, all the algorithms have to evaluate the sparse coding solution of 62,001 patches for images of size 256×256 or 255,025 patches for images of size 512×512. Then, the restoring patches were averaged in the same the procedure as in [8]. All the experiments are repeated 10 times, and the average results are reported.

**Figure 3:**
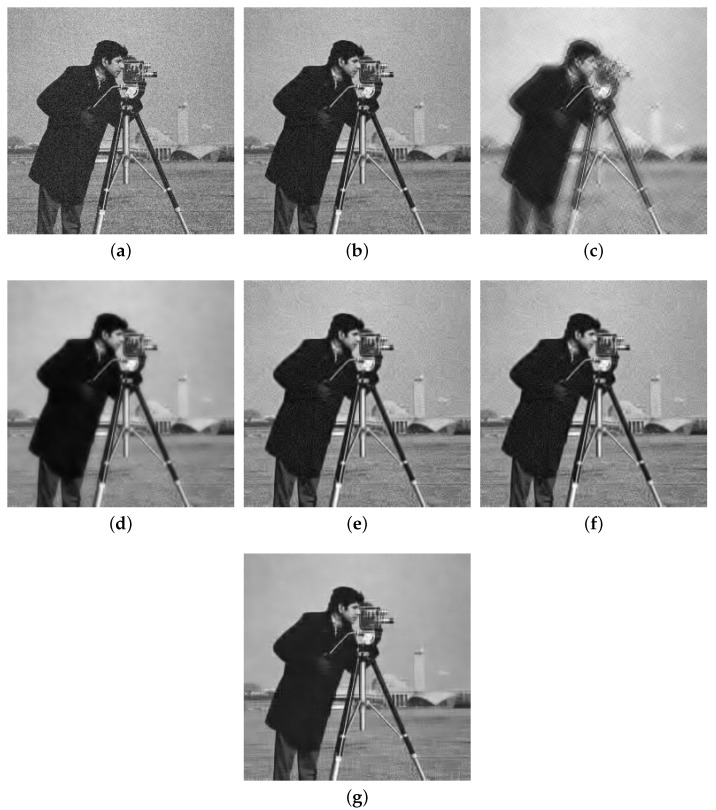
Comparison of the denoising results (PSNR/SSIM) of all the algorithms on the standard cameraman image with σ=15, where s=10. (**a**) Noise image (σ=15); (**b**) OMP (27.62/0.6006); (**c**) SG-HT (22.90/0.7308); (**d**) GraSP (23.50/0.8453); (**e**) SVRGHT (27.96/0.8588); (**f**) LSSG-HT (27.26/0.8433); (**g**) SRGSP (29.08/0.8761).

Table 1 shows the results (including PSNR and SSIM) of all the algorithms at different noise levels, i.e., the values of σ vary from 5 to 55. As we can see from Table 1, our SRGSP algorithm can obtain higher PSNR and SSIM results than the other methods in all the settings, which indicates that the intrinsic low-dimensional structure can be found by our algorithm. Figure 3 shows the visual results of all the methods (i.e., SRGSP, LSSG-HT, SVRGHT, SG-HT, GraSP and OMP) on the cameraman image with σ=15, where s=10. It is clearly visible that the sky region of the cameraman image is well restored by SRGSP, while the results of other methods are not well recovered. Moreover, our SRGSP algorithm has a higher PSNR value of 29.08 dB, compared to 27.96 dB of SVRGHT and 27.32 dB of OMP. The SSIM results of OMP, SVRGHT and SRGSP are 0.6006, 0.8588, 0.8761, respectively. All the above results demonstrate the effectiveness of SRGSP for image denoising tasks.

**Figure 4:**
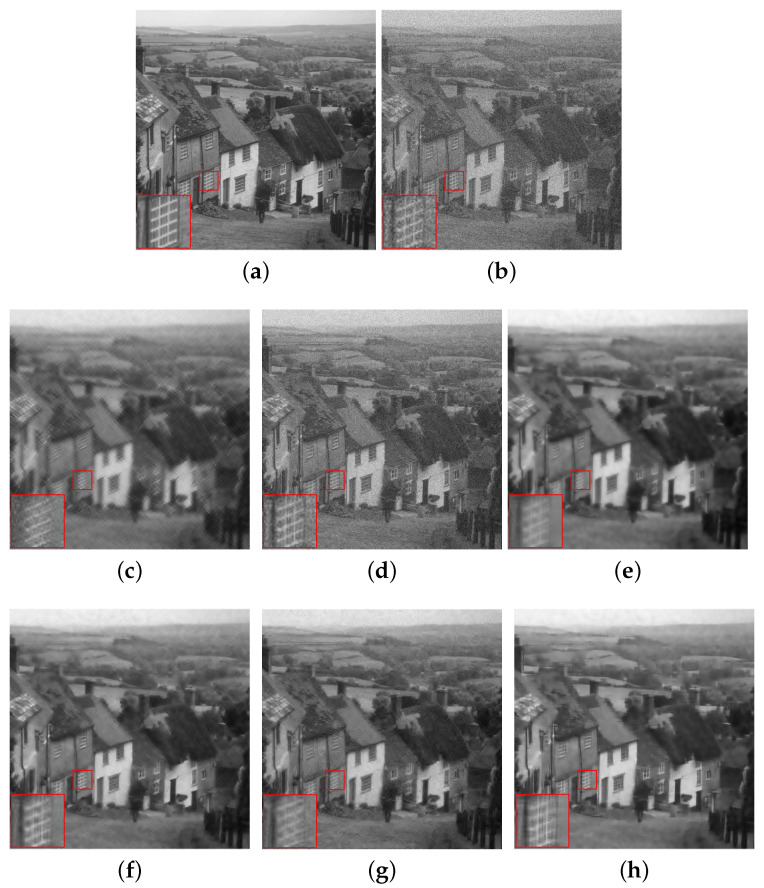
Comparison of the denoising results (PSNR/SSIM) of all the algorithms on the standard hill image with σ=25, where s=10. (**a**) Original image; (**b**) noise image (σ=25); (**c**) SG-HT (25.58/0.5711); (**d**) OMP (21.90/0.4065); (**e**) GraSP (27.01/0.6748); (**f**) SVRGHT (28.16/0.7012); (**g**) LSSG-HT (27.86/0.7095); (**h**) SRGSP (28.53/0.7164).

**Figure 5:**
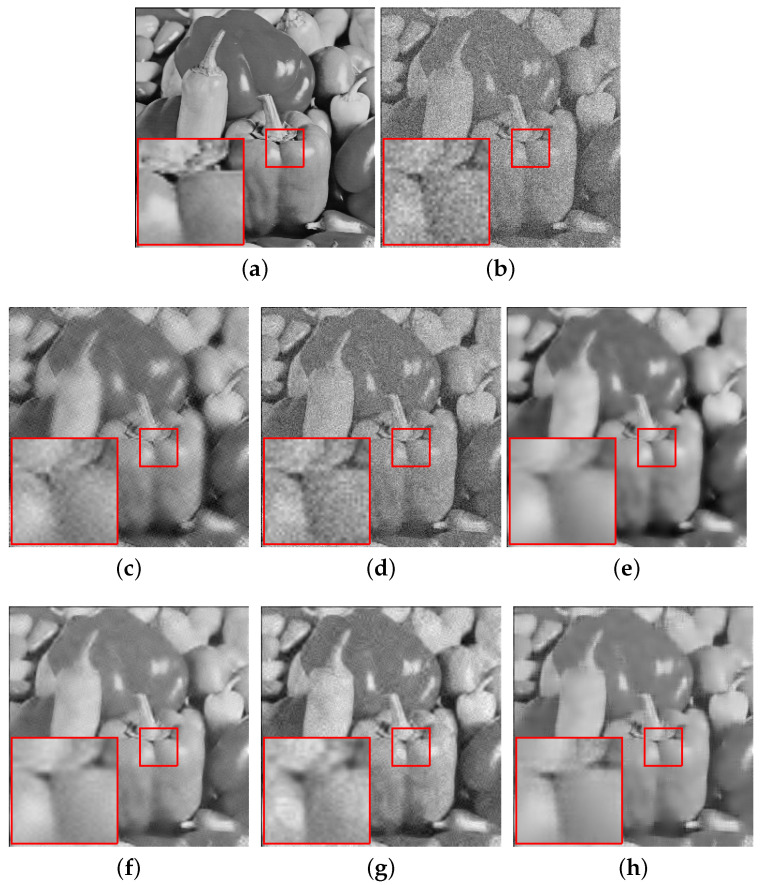
Comparison of the denoising results (PSNR/SSIM) of all the algorithms on the standard peppers image with σ=35, where s=10. (**a**) Original image; (**b**) noise image (σ=35); (**c**) SG-HT (21.65/0.5514); (**d**) OMP (20.48/0.3529); (**e**) GraSP (23.91/0.7546); (**f**) SVRGHT (25.88/0.7390); (**g**) LSSG-HT (25.12/0.7023); (**h**) SRGSP (26.56/0.7851).

**Figure 6:**
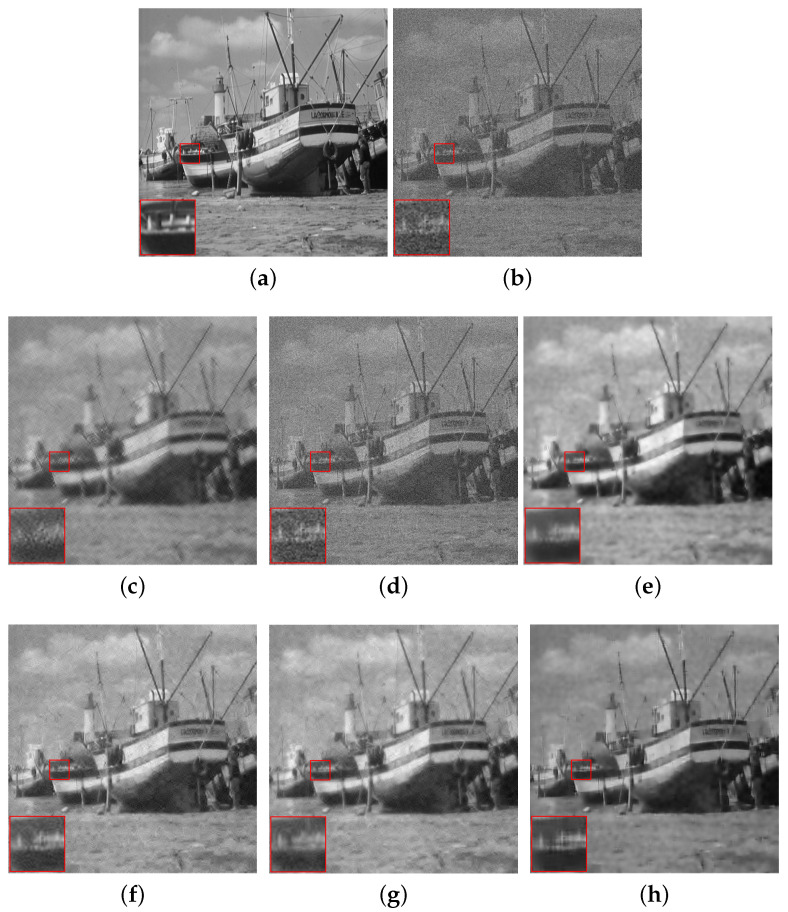
Comparison of the denoising results (PSNR/SSIM) of all the algorithms on the standard boat image with σ=45, where s=10. (**a**) Original image; (**b**) noise image (σ=45); (**c**) SG-HT (22.62/0.4135); (**d**) OMP (16.77/0.2176); (**e**) GraSP (24.42/0.6146); (**f**) SVRGHT (24.68/0.5172); (**g**) LSSG-HT (25.51/0.5321); (**h**) SRGSP (25.68/0.6519).

Figure 4, Figure 5 and Figure 6 show the denoising results of all the algorithms on the hill, pepper and boat images with different noise levels (e.g., σ=25, 35, 45). We can see that our SRGSP algorithm consistently outperforms the other methods in terms of both PSNR and SSIM. Moreover, we give the following suggestion of empirical parameter-tuning for our SRGSP to obtain a good result. Based on all the experimental results, we find that in image denoising tasks, the error bound ϵ can be set empirically to 1.15σ for yielding a good result. For a general parameter setting, the number of outer-iteration *T* is set in the interval [20, 30] and its inter-iteration is set to integer multiples of the number of samples.

### 5.5. Face Recognition

In this subsection, we evaluate the performance of our SRGSP algorithm for face recognition on two real-world face datasets. More specifically, SRGSP is used as the solver in the sparse representation-based classification (SRC) framework [12]. We compare the recognition rates of SRGSP with those of some state-of-the-art sparsity constrained algorithms, such as GraSP, SG-HT, SVRGHT and LSSG-HT. In order to evaluate robustness of our algorithm, we manually add Gaussian noise to the face data.

#### 5.5.1. Datasets

Although there are many datasets available for face recognition, we choose two common publicly datasets, i.e., the AR database [47] and the extended Yale B database [48]. The extended Yale B database contains 2414 frontal-face images of 38 people under different controlled lighting conditions [48]. For each individual, we randomly choose 26 images for training and 15 images for testing. The AR database contains over 4000 color images corresponding to 126 people’s faces (70 Male and 56 Female). The images are obtained under different situation including different facial expressions, illumination conditions, and occlusions such as scarf or sunglasses. For simplicity, we randomly choose 100 objects, and each object has 15 images for training and 11 for testing. Note that the AR database may be difficult for face recognition because there are more classes to recognize and few training samples for each object.

#### 5.5.2. Experimental Setup

For each sparsity constrained algorithm, the sparsity level makes great difference to the solution of sparse representation, especially at different noise levels. Therefore, in order to approach the best performance of all these algorithms, we change the sparsity parameter within a certain range for each algorithm, i.e., s∈[5,10,20,30,40,50]. Thus, we can make sure that all these algorithms achieve the best recognition rates in the parameter setting. In all settings of the experiments, the images are down-sampled to 32×32 pixels. As in [12], a series of processing operations are made to the above datasets. We first rescale the training matrix into [0,1] for the convenience of adding noise, and then add Gaussian noise with zero mean and standard deviation σ. Finally, we normalize the columns of the training matrix to have unit $\ell_{2}$-norm.

#### 5.5.3. Results on Real-World Face Data

**Figure 7:**
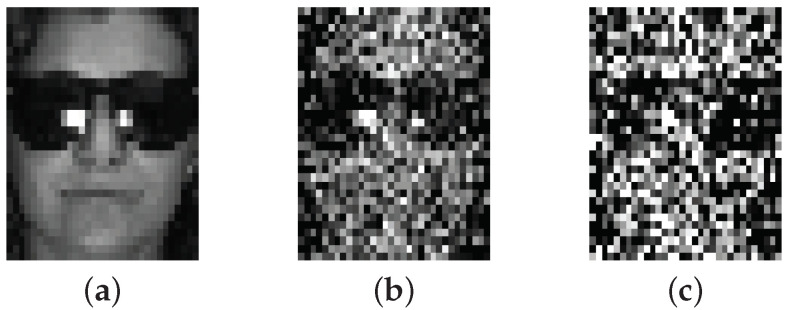
Examples of the AR face database with different levels of white Gaussian noise. (**a**) σ=0; (**b**) σ=0.25; (**c**) σ=0.5.

In this part, we report the recognition rates of SRGSP on both the AR and the extended Yale B databases. Figure 7 shows the real testing images from the AR database with different Gaussian noise levels (e.g., σ=0.25 and σ=0.5). As we can see, it is challenging for humans to correctly recognize the face images under this situation. However, even in this extreme conditions, SRGSP achieves a high recognition rate with high probability.

**Figure 8:**
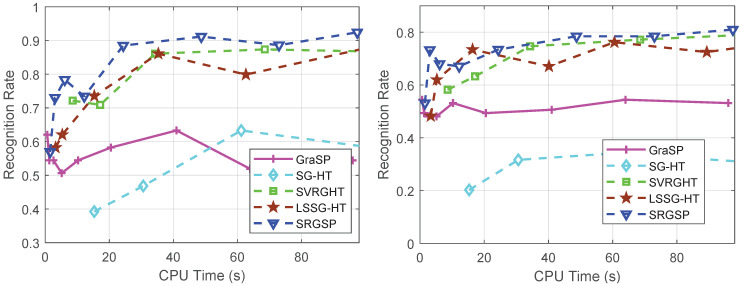

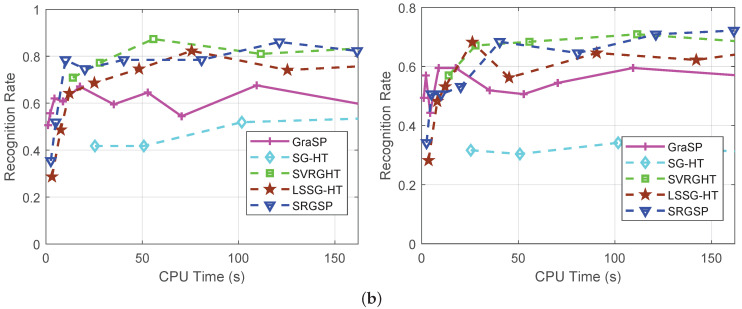
Recognition rates of all the algorithms on the extended Yale B (top) and AR (bottom) databases with different levels of Gaussian noise: σ=0.25 and σ=0.5. (**a**) The Yale B database with σ=0.25 (left) and σ=0.5 (right); (**b**) the AR database with σ=0.25 (left) and σ=0.5 (right).

**Figure 9:**
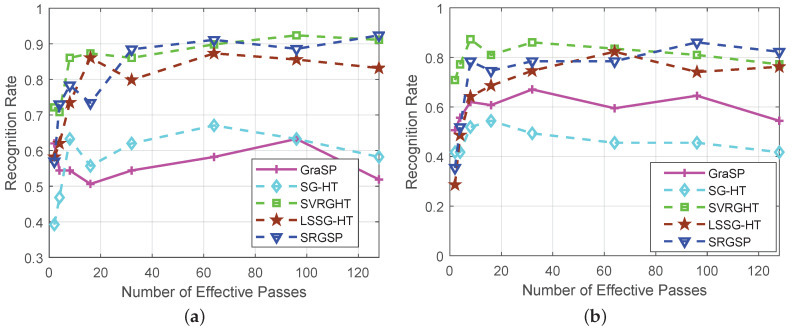
Comparison of recognition rates of all the algorithms on the extended Yale B and AR databases with the Gaussian noise level σ=0.25. (**a**) The Yale B dataset; (**b**) the AR dataset.

**Figure 10:**
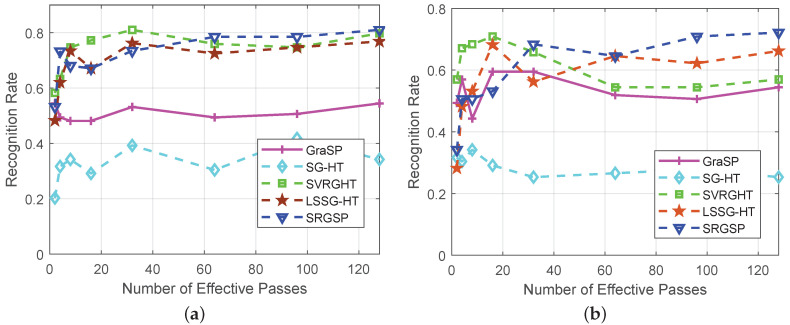
Comparison of recognition rates of all the algorithms on the extended Yale B and AR databases with the Gaussian noise level σ=0.5. (**a**) The Yale B dataset; (**b**) The AR dataset.

In Figure 8, SVRGHT, LSSG-HT and SRGSP obtain much higher recognition rates than other two methods on both datasets, which verifies the superiority of variance reduction or recursive gradient techniques. Although at the very early iterations, SRGSP may have slightly lower recognize rates than SVRGHT, while with the increase of iterations, SRGSP can achieve the highest recognition rate among all the classifiers. Moreover, SRGSP is several times faster than SVRGHT due to less hard thresholding operations. For example, when σ=0.25 on the Yale B database, SRGSP obtains over 90% recognition rate within 50 s, while SVRGHT needs more CPU time to reach the same accuracy. In fact, in the same number of passes, SRGSP still achieves the highest recognition rate among the state-of-the-art hard thresholding algorithms, which demonstrates the effectiveness and efficiency of SRGSP. In the extreme situation of the AR database with the Gaussian noise level σ=0.5, SRGSP still achieves a higher recognition rate than the other methods. The results of recognition rates with respect to the number of passes on both datasets are provided in Figure 9 and Figure 10, and further demonstrate the superiority of SRGSP.

## 6. Conclusions and Future Work

In this paper, we proposed a stochastic recursive gradient support pursuit (SRGSP) method for solving large-scale sparsity constrained optimization problems. We also provided the convergence analysis of SRGSP, which shows that SRGSP obtains a linear convergence rate. As existing hard thresholding-based algorithms need more thresholding operations, SRGSP just needs a hard thresholding operation in each epoch, and thus has a significantly per-iteration lower computational complexity, i.e., O(d) vs. O(dlog(d)). Experimental results on synthetic and real large-scale datasets verified the effectiveness and efficiency of SRGSP.

Moreover, we also applied our SRGSP method to tackle image denoising and face recognition tasks, where sparse representation learning plays an important role. Our experimental results show that SRGSP outperforms other sparse representation methods in terms of PSNR and recognition rates. Note that for the image denoising application, the dictionary is the fixed overcomplete DCT dictionary. Inspired by some sophisticated methods such as K-SVD [38], we will iteratively update the dictionary in the future, which can further improve performance. In fact, there are many real-world sparse representation learning applications such as image super-resolution, image restoration, image classification and visual tracking. Therefore, we will apply our SRGSP method to address more applications in the future. In addition, our SRGSP algorithm can be extended to tackle low-rank matrix and tensor completion and recovery problems as in [49,50,51].

## Figures and Tables

**Table 1:** The denoising results (PSNR (dB)/SSIM) of all the methods including OMP [46], GraSP [22], SG-HT [23], SVRGHT [24], LSSG-HT [34] and our SRGSP method on 6 standard images at different noise levels from 5 to 55. The best performance is shown in bold.

σ	Algorithms	Peppers	Cameraman	House	Man	Hill	Boat
5	SRGSP	**34.45/0.9259**	**34.12/0.9195**	**35.64/0.9322**	**34.15/0.9149**	**34.25/0.9132**	**34.68/0.9256**
	LSSG-HT	33.56/0.9022	32.65/0.8932	34.12/0.9123	33.26/0.8865	33.35/0.8995	33.32/0.9203
	SVRGHT	33.95/0.9135	33.25/0.9065	34.01/0.9023	33.95/0.8997	33.39/0.9012	33.61/0.9165
	SG-HT	25.56/0.7801	26.62/0.7725	27.56/0.7835	27.65/0.7832	27.85/0.7532	25.89/0.7710
	GraSP	27.89/0.8832	24.72/0.8632	30.85/0.8857	28.25/0.7755	28.65/0.8278	26.91/0.7897
	OMP	34.02/0.8932	33.25/0.8706	34.23/0.8769	32.35/0.8562	33.35/0.8623	33.73/0.9143
10	SRGSP	**32.64/0.8942**	**32.05/0.8867**	**33.95/0.8932**	**32.13/0.8549**	**32.85/0.8535**	**32.56/0.8691**
	LSSG-HT	31.68/0.8822	30.22/0.7823	33.15/0.8734	31.56/0.8305	31.56/0.8462	31.98/0.85.65
	SVRGHT	31.95/0.8835	29.56/0.7656	33.05/0.8721	31.35/0.8497	31.12/0.8342	31.56/0.8479
	SG-HT	24.48/0.7532	24.98/0.7272	26.85/0.7373	26.95/0.7326	26.89/0.7265	25.56/0.7235
	GraSP	27.56/0.8596	24.25/0.8323	30.15/0.8657	27.65/0.7651	28.26/0.7578	26.54/0.7589
	OMP	30.25/0.7656	29.56/0.7685	29.52/0.7685	28.36/0.7265	29.65/0.7552	29.89/0.7551
15	SRGSP	**30.57/0.8759**	**29.10/0.8707**	**32.81/0.8622**	**30.34/0.8249**	**30.65/0.7990**	**30.53/0.8191**
	LSSG-HT	29.95/0.8432	27.26/0.7102	32.12/0.8234	29.96/0.8105	30.24/0.7895	30.35/0.7956
	SVRGHT	30.35/0.8585	27.84/0.6927	32.26/0.8513	30.09/0.8197	30.34/0.7897	30.34/0.8079
	SG-HT	23.07/0.7311	22.92/0.6872	25.53/0.7073	26.12/0.7066	26.57/0.6721	24.89/0.6635
	GraSP	27.04/0.8449	23.46/0.8086	29.65/0.8357	26.97/0.7455	27.87/0.7178	26.11/0.7207
	OMP	27.76/0.6602	27.32/0.6006	27.80/0.5769	27.69/0.6504	27.70/0.6592	26.37/0.6051
25	SRGSP	**28.19/0.8232**	**27.37/0.8186**	**30.39/0.8224**	**28.10/0.7470**	**28.53/0.7164**	**28.18/0.7445**
	LSSG-HT	27.35/0.7785	26.35/0.5121	29.56/0.7806	27.85/0.7531	27.86/0.6842	27.96/0.7095
	SVRGHT	27.85/0.7606	26.85/0.5232	29.48/0.7797	27.92/0.7323	28.09/0.6998	27.82/0.7012
	SG-HT	22.34/0.6386	22.40/0.5810	24.68/0.5974	25.15/0.6030	25.57/0.5757	24.13/0.5711
	GraSP	26.12/0.8078	24.66/0.7715	28.50/0.8034	27.09/0.7188	27.00/0.6617	25.54/0.6748
	OMP	23.40/0.4696	23.23/0.4304	23.35/0.3838	23.30/0.4458	23.31/0.4457	21.91/0.4065
35	SRGSP	**26.55/0.7851**	**25.84/0.7674**	**28.62/0.7909**	**26.84/0.6966**	**27.29/0.6642**	**26.74/0.6920**
	LSSG-HT	25.16/0.7023	25.32/0.6812	27.65/0.7126	26.48/0.6610	26.56/0.6215	26.25/0.6126
	SVRGHT	25.94/0.7390	25.46/0.6907	27.46/0.7046	26.52/0.6627	26.85/0.6371	26.05/0.6025
	SG-HT	21.68/0.5514	21.77/0.4840	23.78/0.4916	24.28/0.5159	24.68/0.4950	23.37/0.4870
	GraSP	23.96/0.7546	22.91/0.7080	26.33/0.7549	25.67/0.6639	26.29/0.6269	25.02/0.6442
	OMP	20.46/0.3529	20.24/0.3264	20.40/0.2780	20.42/0.3215	20.38/0.3121	18.94/0.2882
45	SRGSP	**25.26/0.7501**	**24.76/0.7258**	**27.53/0.7601**	**25.83/0.6575**	**26.40/0.6247**	**25.71/0.6519**
	LSSG-HT	24.05/0.6502	24.12/0.6321	26.64/0.6532	25.32/0.5962	25.62/0.5933	25.51/0.5321
	SVRGHT	24.44/0.6722	24.24/0.6151	26.28/0.6375	25.46/0.6009	25.95/0.5807	24.71/0.5172
	SG-HT	21.05/0.4754	21.24/0.457	23.04/0.4120	23.38/0.4386	23.80/0.4207	22.63/0.4135
	GraSP	23.30/0.7264	22.70/0.6848	25.76/0.7330	25.03/0.6355	25.71/0.6014	24.43/0.6146
	OMP	18.27/0.2724	18.20/0.2604	18.24/0.2134	18.20/0.2384	18.18/0.2261	16.77/0.2176
55	SRGSP	**24.28/0.7254**	**23.90/0.6946**	**26.28/0.7203**	**25.16/0.6271**	**25.74/0.5988**	**24.84/0.6179**
	LSSG-HT	23.35/0.6212	23.01/0.5621	25.43/0.5963	24.52/0.5632	25.01/0.5423	24.32/0.4623
	SVRGHT	23.62/0.6183	23.22/0.5519	25.23/0.5708	24.74/0.5480	25.27/0.5349	23.61/0.4462
	SG-HT	20.51/0.4127	20.60/0.3484	22.25/0.3449	22.59/0.3774	22.98/0.3635	21.91/0.3546
	GraSP	22.86/0.7050	22.36/0.6646	25.04/0.7050	24.57/0.6136	25.30/0.5854	23.94/0.5918
	OMP	16.50/0.2203	16.43/0.2174	16.44/0.1665	16.48/0.1852	16.47/0.1720	15.02/0.1667

## Appendix A

In this paper, we use $A\;=\;\left\{a_{1},a_{2},\ldots ,a_{n}\right\}\;\in \;R^{d\times n}$ to denote the design matrix, $y\;=\;{\left[y_{1},y_{2},\ldots ,y_{d}\right]}^{T}\;\in \;R^{d}$ to denote the response vector and $x\;=\;{\left[x_{1},x_{2},\ldots ,x_{n}\right]}^{T}\;\in \;R^{n}$ to denote the model parameter. For the parameter vector $x\;\in \;R^{n}$, ${∥x∥}_{2}$ denotes its $\ell_{2}$-norm, while ${∥x∥}_{1}$ is the $\ell_{1}$-norm.

### Sparse Representation-Based Classification (SRC)

In this paper, we apply our algorithm into the SRC framework for face recognition tasks, as outlined in Algorithm A1.

**Algorithm A1**: Sparse Representation-based Classification
Input: A matrix of training samples $A\;=\;\left[A_{1},A_{2},\ldots ,A_{k}\right]\;\in \;R^{d\times n}$ for *k* classes, sparsity parameter *s*, and a test sample $y\in R^{d}$. 1: Normalize the columns of *A* to have unit $\ell_{2}$-norm; 2: Solve the $\ell_{0}$-minimization problem: $\hat{x}={\arg\min∥y-Ax∥}_{2}^{2},\;\;s.t.,\;{∥x∥}_{0}\leq s$; 3: Compute the residuals $r_{i}\left(y\right)={∥y-A\delta_{i}\left(\hat{x}\right)∥}_{2}$ for i=1,⋯,k; Output: identity $\left(y\right)=\arg\min r_{i}\left(y\right)$.
